# Physical Activity Levels among Preschool-Aged Children in Family Child Care Homes: A Comparison between Hispanic and Non-Hispanic Children Using Accelerometry

**DOI:** 10.3390/children8050349

**Published:** 2021-04-28

**Authors:** Augustine W. Kang, Kim M. Gans, Tayla von Ash, Danielle Castagneri, Laura Dionne, Alison Tovar, Patricia M. Risica

**Affiliations:** 1 Department of Behavioral and Social Sciences, Brown University School of Public Health, Providence, RI 02912, USA; kim_gans@brown.edu (K.M.G.); tayla_ash@brown.edu (T.v.A.); Danielle_castagneri@alumni.brown.edu (D.C.); Laura_dionne@brown.edu (L.D.); patricia_risica@brown.edu (P.M.R.); 2 Department of Human Development and Family Studies, University of Connecticut, Storrs, CT 06269, USA; 3 Department of Nutrition and Food Sciences, University of Rhode Island, Kingston, RI 02881, USA; alison_tovar@uri.edu; 4 Department of Epidemiology, Brown University School of Public Health, Providence, RI 02912, USA

**Keywords:** physical activity, family child care homes, health disparities, hispanic

## Abstract

Obesity prevalence among Hispanic children is twice that of non-Hispanic white children; Hispanic children may also engage in less physical activity (PA) compared to non-Hispanic white children. A large number of U.S. preschool-aged children are cared for in Family Child Care Homes (FCCH), yet few studies have examined PA levels and ethnicity differences in PA levels among these children. We examine baseline data from a cluster-randomized trial (Healthy Start/Comienzos Sanos) to improve food and PA environments in FCCHs. Children aged 2-to-5-years (*n* = 342) wore triaxial accelerometers for two days in FCCHs. Variables examined include percentage of time (%) spent in sedentary, and light, moderate, and vigorous PA. The full dataset (*n* = 342) indicated sedentary behavior 62% ± 11% of the time and only 10% ± 5% of the time spent in moderate-to-vigorous PA. Among children in the upper-median half of wear-time (*n* = 176), Hispanic children had significantly greater % sedentary time vs. Non-Hispanic children (66.2% ± 8.3% vs. 62.6% ± 6.9%, *p* = 0.007), and lower % light PA (25.4% ± 6.3% vs. 27.7% ± 4.9%, *p* = 0.008) and moderate PA (5.5% ± 2.1% vs. 6.4% ± 2.2%, *p* = 0.018). Our results highlight that PA levels were lower among our sample compared to previous studies, and that Hispanic children were more sedentary and less active compared to non-Hispanic white children.

## 1. Introduction

Childhood obesity has more than tripled since the 1970s and currently affects 13.9% of preschool-aged (2-to-5-year old) children [1,2]. Racial/ethnic disparities exist such that the prevalence of obesity among Hispanic children (23.6%) is almost double the prevalence compared to non-Hispanic White children (13.5%) [1]. Prevention of childhood obesity is critical given that children who are overweight/obese have an increased risk of developing chronic diseases (e.g., diabetes mellitus and cardiovascular disease), and these risks track into adulthood [3]. Although the etiology of obesity is complex, sedentary behaviors and inadequate levels of physical activity (PA) are contributing factors to childhood obesity [4].

Regular PA among children also presents a myriad of health benefits independent of BMI control (including lowered blood lipids, adiposity, improved bone mineral density, and cognitive development [5]), attenuating the risks of obesity-associated chronic diseases [3,4,5]. World Health Organization guidelines on PA and sedentary behavior for children recommend: (1) 180 min of PA (at any intensity) for 2-year old children, (2) 180 min of PA, of which 60 min are moderate-to-vigorous physical activity (MVPA) for 3-to-4-year old children, and (3) at least 60 min of MVPA for 5-year old children [6,7]. Studies have reported that only 25% of all children and youth in the U.S. currently meet the recommended amounts of PA (i.e., 60 min of moderate to vigorous physical activity, or MVPA, for at least 3 days per week) [8]. Among preschool-aged children, less than 60 min of MVPA per day was reported by nearly half of the studies in a systematic review of PA in preschool-aged children [9]. Thus, researchers have called for more studies examining PA levels among young children across environments, such as childcare settings [10].

Societal shifts in the workforce have resulted in an increasing demand for childcare. In the U.S., approximately 61% (12.5 million) of children receive non-parental childcare outside the home [11]. In addition, almost 2 million (or 25%) preschool-aged children in the U.S. are cared for in Family Child Care Homes (FCCH). FCCHs represent unique settings in which to examine PA levels among children as they are both childcare settings and providers’ homes. In terms of the PA context, FCCHs mainly differ from mainstream childcare centers in that FCCHs tend to have greater logistical and space constraints than centers, which may limit the amount and type of PA that children have access to. However, few studies have examined PA levels in FCCHs, and to our knowledge, none have examined ethnic differences in PA levels among children in FCCHs.

Therefore, the goal of this study is to fill critical gaps in the literature by documenting the levels of objectively measured PA among preschoolers in the context of FCCHs. Additionally, we seek to leverage an ethnically diverse sample by examining differences in PA levels among preschool-aged children by ethnicity (specifically Hispanic vs. non-Hispanic children).

## 2. Materials and Methods

This study examines baseline data from a cluster randomized controlled trial, Healthy Start/Comienzos Sanos, which studies the efficacy of a multicomponent intervention to improve the food and physical activity environments in FCCHs and the diet, PA, and screen time behaviors of 2-to-5-year old children in their care [12]. Data was collected between 2017 and 2018 and was analyzed in 2019. A total of 119 FCCHs from Rhode Island and Massachusetts were enrolled; within these homes, *n* = 342 children ages 2-to-5-years old completed baseline accelerometer measurements. FCCHs were recruited within 60 miles of the Providence, Rhode Island metropolitan area. Consent to measure was obtained from the children’s parents; assent was obtained from the participating children. This study was approved by the Institutional Review Boards of Brown University, University of Connecticut, and The University of Rhode Island.

### 2.1. Demographics and Anthropometrics

Children’s demographic information (specifically, age, sex, race, ethnicity) was obtained from a parent-reported survey. Standard techniques were used to measure the height, weight, and waist circumference of participating children in accordance with CDC NHANES protocol [13,14]. All three measurements were repeated a total of 3 times and averaged for each child. Age- and sex-adjusted BMI z-scores were calculated from the obtained height and weight information.

### 2.2. Accelerometry

Triaxial GT3X^TM^ ActiGraph (ActiGraph LLC, Pensacola, FL, USA) accelerometers were used to measure children’s PA levels. The use of accelerometry has been validated among the pediatric population [15]. The accelerometer was affixed at the child’s right hip on a Velcro (Velcro companies, Manchester, NH, USA) belt during their time at childcare over the two-day observation period. FCCH provider-initiated naptimes were recorded by observers. Five-second epochs were used to better detect short bursts of activity, characteristic of PA in 2-to-5-year old children [16]. Cut-points were used in accordance with Freedson et al.’s recommendations for 2-to-5-year old children to categorize activity into graduated levels (sedentary, light, moderate, vigorous, and moderate-to-vigorous [MVPA]) based on the metabolic equivalent of tasks (METs) and Butte Preschoolers vector magnitude (2013) were used [17,18]. In addition, we included naptimes in our analysis because all FCCHs in our study scheduled a naptime (and children kept their accelerometers worn during those times). As the children were required to be sedentary, we adopted a uniform approach of including all nap time in our analysis [19].

### 2.3. Accelerometry Dataset Manipulation

For studies attempting to measure PA among young children over a 24-h period, a wear time of at least 10 h during wake time using triaxial accelerometers is recommended [20]. Due to time constraints in the FCCH setting, the average ActiGraph wear time per day in our study was 5.6 h (range 1 h to 9.5 h), and the median wear time was 6.3 h. As we were mainly focused on measuring PA during the time the child spent at the FCCH and not the entire day (i.e., at home), these wear times should be adequate to capture PA during the hours when the child is at the FCCH. We also created two sets of accelerometer datasets: the full dataset regardless of wear time, and another focusing on participants in the upper median-half of wear time to examine if longer wear times influenced the differences in the detection of PA levels and between-ethnicity differences in PA. We present data for both the upper-median half of accelerometer wear-time and the full sample, as well as information for PA levels as a percentage of total wear time and not minutes per hour (min/hour) due to the highly variable wear time among participants, and that children did not wear the accelerometers for a full 24-h period.

### 2.4. Statistical Analysis

Exploratory data analysis examined the dataset for normality, outliers, and missing data. Frequencies and descriptive statistics of participant demographics, anthropometrics, and PA levels were examined and presented. One-way ANOVA was used to detect between-group differences in age, sex, and between-ethnicity differences in PA levels, with Tukey’s post hoc tests used for follow-up pairwise comparisons. Chi-square analysis was used to examine between-group differences between ethnicity and categorical variables. Significant univariate associations were subsequently controlled for in multivariate ANCOVA when examining between-ethnicity differences. α was set at 0.05. All analyses were repeated for the two datasets for full wear time and upper median-half of wear time (median = 6.3 h; *n* = 342 and *n* = 176, respectively). All analyses were completed using the Statistical Package for Social Sciences, Version 24 [21].

## 3. Results

### 3.1. Demographics

The mean age of the study sample was 41.5 months; of the full sample (*n* = 342), 36% were 2 years old, 31% were 3 years old, and 33% were 4–5 years old. Over half (52%) of participating children were female, and 56% were Hispanic. More than two thirds (74%) of the children were White, and 11% were multi-racial. One-third (33%) of the sample were overweight (85th to 94th percentile) or obese (≥95th percentile) based on sex- and age-adjusted BMI Z-score standards. Demographics of the upper-median half group did not significantly differ from the full sample (Table 1).

### 3.2. Physical Activity Levels—Complete Cases

Using complete-cases from our accelerometry dataset, PA levels of the study sample measured: 61.5% sedentary behavior; 26.5% light PA; 6.4% moderate PA; 3.5% vigorous PA, and; 9.9% MVPA. There were significant between-group differences in age groups on moderate PA (*p* < 0.001), vigorous PA, (*p* < 0.001), and MVPA (*p* < 0.001), where 4-to-5-year olds had significantly higher measured PA levels compared to 2-year-olds and 3-year-olds. Boys also had significantly higher levels of moderate PA (*p* = 0.013), vigorous PA (*p* = 0.003), and MVPA (*p* = 0.003) than girls (Table 2).

### 3.3. Physical Activity Levels—Upper-Median Half of Wear-Time

In examining the subset of children in the upper-medial half of accelerometer wear-time in our dataset, the PA levels of the study sample (*n* = 176) measured: 64.6% sedentary behavior; 26.5% light PA; 5.9% moderate PA; 3.0% vigorous PA, and; 8.9% MVPA. There were significant differences between age groups on moderate PA (*p* < 0.001), vigorous PA, (*p* < 0.001), and MVPA (*p* < 0.001), where 3-year-olds and 4-to-5-year olds had significantly higher PA levels compared to 2-year-olds. There were also significant differences in sedentary behavior (*p* = 0.033), where 2-year-olds had significantly higher sedentary behavior than 4-to-5-year olds. Boys also had significantly higher levels of moderate PA (*p* = 0.007), vigorous PA (*p* = 0.001), and MVPA (*p* = 0.002) than girls (Table 3).

### 3.4. Between-Ethnicity Comparisons: Hispanics versus Non-Hispanics—Complete Cases

Hispanic and Non-Hispanic children did not differ in any of the demographic or anthropometric variables, except that Hispanic children had significantly larger waist circumferences (53.6 cm) compared to non-Hispanic children (52.2 cm), *p* = 0.035. There were no statistically significant differences between Hispanic and Non-Hispanic children in PA (Table 4).

### 3.5. Between-Ethnicity Comparisons: Hispanics versus Non-Hispanics—Upper-Median Half of Wear-Time

Hispanic children spent a significantly greater percent of their time sedentary (66.2%) compared to non-Hispanic children (62.6%), *p* = 0.007. Hispanic children also spent a significantly lower percent of their time in light PA (25.4%) compared to non-Hispanic children (27.7%), *p* = 0.008. In addition, Hispanic children spent a significantly lower percent of their time in moderate PA (5.5%) compared to non-Hispanic children (6.4%), *p* = 0.018. Lastly, there was a marginally significant difference between Hispanic and non-Hispanic children in percent of time spent in MVPA; with Hispanic children spending a slightly lower percent of their time in MVPA (8.4%) compared to non-Hispanic children (9.6%), *p* = 0.085 (Table 4).

## 4. Discussion

Given the lack of studies examining PA levels among children in FCCHs, the goal of our study was to document the prevalence of objectively measured PA levels among preschoolers in FCCHs and compare ethnicity differences in PA levels. Overall, our study found low levels of PA among 2-to-5-year old children in FCCHs, similar to previous studies [22,23]. In addition, our study also found between-sex, between-age group, and between-ethnicity differences in PA levels. To our knowledge, this is the first study to examine differences in PA levels across ethnic groups in FCCHs.

Mean BMI and prevalence of overweight in our study sample were higher than national estimates; the average BMI percentile in our sample was 66.5, with 33% of our sample classified as overweight/obese, compared to national estimates of 26% overweight/obese prevalence in this age group [24,25]. While Hispanic children were not more likely to be overweight than non-Hispanic children in our sample, we did find that Hispanic children had a significantly higher waist circumference than non-Hispanic White children.

Variable estimates of PA levels have been found across different studies of preschool-aged children, in part due to variation in instruments and methods, including the use of different accelerometer cut-points to classify PA levels. Regardless of methods, high levels of sedentary behavior and low levels of MVPA among preschoolers have been consistently reported. Preschoolers were estimated to spend only about 5.5% of their time in MVPA per day, according to a meta-analysis pooling the accelerometry-measured estimates of MVPA from 29 studies of more than 6000 preschool-age children (3–5 years) [19]. Most of these studies were conducted in home-based, not childcare-based settings.

The childcare setting is important in influencing children’s PA levels [26]. A review of PA studies in childcare centers reported that while available studies are inconsistent in their approach to PA measurement, there appears to be a consistent trend of high rates of sedentary behavior in childcare centers [27]. While few studies have examined PA levels in FCCH, those that did report that children do not obtain sufficient PA relative to national guidelines [22,28,29,30]. While our study results indicate low levels of PA similar to previous studies, we identified lower MVPA levels (i.e., 5.3 ± 2.8 min/hour for the naptime-included dataset (full sample, data not presented in Section 3) compared to other studies examining PA among children in FCCHs. For example, Delaney et al. (2014) reported 8.8 ± 2.6 min/hour MVPA [22,23]; Rice & Trost (2014) reported 5.8 ± 3.2 min/hour MVPA) [22,23]. In contrast, another study examining PA in FCCHs reported only 1.76 ± 0.90 min/hour MVPA among their sample [29]. PA practices may vary considerably between FCCHs, due in part to differing individual provider practices and a highly variable home environment that may or may not facilitate PA for children [31]. A recent study found that the amount of indoor space available for active play was associated with MVPA [32]. Hence, a potential way to increase PA in FCCHs could be to target the physical environment, such as providing additional space and equipment, or creative ways to arrange the space to offer more room for active play. Provider training might also focus on the importance of moderate and vigorous PA both indoors and outdoors.

Consistent with the literature, we also found that PA levels were higher for boys than for girls [33]. Potential explanations for gender disparities in PA include social norms, such as gender socialization (that engaging in PA is more appropriate for boys). Girls have also been reported to receive less support and encouragement to engage in PA [15,16]. As discussed by Telford et al. (2016), strategies to improve gender disparities in childcare settings should be multicomponent (by targeting individual/family/school/environment factors) and involve a comprehensive approach to increase PA engagement among boys and girls [17].

Previous studies report that Hispanic children of all ages engage in lower levels of PA than non-Hispanic children [18,34,35], though results among younger children have been mixed [36]. Differences in PA assessment methods and covariate adjustment (e.g., age, sex, and socioeconomic status) may explain differential findings across studies. In our study, we found that Hispanic children had significantly lower light/moderate PA and higher sedentary behavior compared to non-Hispanic children only when restricting analyses to the upper half of the accelerometer wear time. Studies of accelerometry as a measurement tool have shown that increased wear time reduces noise and increases the effect size and reliability of PA measurement [37]. Hence, our study results suggest that increased wear time allowed us to obtain a larger effect size, which may have led to the detection of between-group differences.

Lower PA levels are associated with an increased risk for obesity and cardiovascular disease among children [25,38]. While results from studies examining the association between PA and body mass composition among preschool-aged children remain mixed, reviews of available evidence suggest that PA levels in early childhood may track into adulthood [35,39]. Racial/ethnic gaps in PA levels between Hispanic and Black children compared to White children are widening [40]. Differences in risk for overweight across race/ethnicity also appear to increase over time, with disparities in obesity present by age 4 [41,42]. Thus, it is plausible that efforts to increase PA levels among preschool-aged children may at least in part ameliorate differences in obesity rates and related adverse health outcomes across race/ethnicity. Given these increasing disparities, as well as the evidence consistently demonstrating the benefits associated with PA (e.g., improved cognitive functioning and mental health), further investigations of PA levels across race/ethnicity in preschool are warranted [43,44]. In particular, interventions seeking to provide better training of FCCH providers are needed; interventions should seek to address barriers such as cultural-based attitudes about activity and the lack of physical infrastructure for PA in FCCHs and the difficulty of working with both FCCH providers and parents in providing consistent messages across home and FCCH environments [45].

While findings from our study are informative, the interpretation of results should be made with several limitations in mind. The cross-sectional design of our study limits our ability to draw conclusions regarding the causality and directionality of observed associations. While the use of accelerometry is a reliable and objective method of assessing PA, our study measured PA over a two-day period, with an average and median wear-time of 5.6 and 6.3 h, respectively (hence falling short of the 3-day, 10-h daily minimum guidelines recommended by Penpraze et al. (2006) [20]). We addressed wear-time issues to some extent by examining the upper-median half of the wear-time, but it is important to recognize that wear-time in the FCCH setting is constrained by factors such as pick-up and drop-off time by parents. In addition, PA data in our study was reported in terms of percentage wear time, which differs from other studies in the literature reporting PA among children (a methodological decision made in consideration of the lower wear time among our sample). However, considering the dearth of data examining PA among FCCHs, overall our study results fill important research gaps.

## 5. Conclusions

We report low levels of MVPA overall, and lower PA for younger children and girls compared to older children and boys. We also report significant differences in levels of PA and sedentary behavior between ethnic groups with Hispanic children having lower PA. These observed associations suggest the importance of further research to determine the reasons for these differences (e.g., FCCH PA opportunities) and possibly the development of tailored interventions to promote PA among ethnically diverse preschool-aged children as part of obesity prevention efforts. In addition, in the FCCH setting, the examination of actual child activities, provider practices, and their association with objectively measured PA levels are needed as provider-led play is a predictor of child PA. Innovative ways to train and provide support for FCCPs are needed to increase MVPA in FCCHs, and community-based, multicomponent intervention approaches should be considered as intervention delivery methods.

## Figures and Tables

**Table 1:** Participant Characteristics.

	All	Upper-Half of Median Accelerometer Wear Time	
	(*n* = 342)	(*n* = 176)	*p*
*Age (Months)*	41.5 ± 11.8	40.8 ± 11.3	0.543
*Age (Years)*			0.856
2-year olds	122 (36)	67 (38)	
3-year olds	105 (31)	53 (30)	
4–5-year olds	115 (33)	56 (32)	
*Sex*			0.989
Female	179 (52)	92 (52)	
Male	163 (48)	84 (48)	
*Ethnicity*			0.329
Hispanic	194 (56)	91 (51)	
Non-Hispanic	140 (44)	79 (49)	
*Race*			0.843
White	254 (74)	133 (74)	
Black	32(9)	18 (11)	
Asian	3 (1)	2 (1)	
American Indian/Native Hawaiian	6 (2)	1 (1)	
>1 race	36 (11)	18 (10)	
Don’t Know/Refused	11 (3)	4 (3)	
Weight Status *			0.230
Non-Overweight	214 (63)	121 (68)	
Overweight	113 (33)	50 (32)	
*BMI z-score Distribution*			0.284
1st to 16th Percentile (>1SD below M)	25 (8)	14 (8)	
17th to 50th Percentile (1SD below M to M)	67 (20)	39 (25)	
51st to 84th Percentile (M to 1SD above M)	122 (35)	68 (35)	
85th to 99th Percentile (>1SD above M)	113 (33)	50 (32)	
*Waist circumference (cm)*			0.522
2-year olds	50.3 ± 4.6	50.5 ± 5.1	
3-year olds	52.6 ± 4.3	52.8 ± 4.6	
4–5-year olds	56.4 ± 5.6	54.8 ± 4.3	

Note. Data expressed as M ± SD or *n* (%). * Based on BMI z-score (<1 standard deviation = Non-overweight; ≥1 standard deviation = overweight); Overweight includes obese children. *n* = 8 missing data for ethnicity; *n* = 15 missing data for weight. Cases with missing data were excluded from analysis.

**Table 2:** Overall Frequencies of Physical Activity and the Association with Age and Sex (*n* = 342).

	%Sedentary	*p*	%Light	*p*	%Moderate	*p*	%Vigorous	*p*	%MVPA	*p*
Overall	61.5 ± 11.5	NA	26.5 ± 5.8	NA	6.4 ± 2.7	NA	3.5 ± 2.4	NA	9.9 ± 4.7	NA
*Age (Years)*		0.083		0.087		**<0.001**		**<0.001**		**<0.001**
2-year olds	61.8 ± 11.3		29.4 ± 8.1 ^2^		5.8 ± 2.5 ^3^		2.9 ± 2.8 ^3^		8.8 ± 4.7 ^3^	
3-year olds	63.0 ± 11.5 ^3^		27.0 ± 8.2 ^1^		6.3 ± 2.8 ^3^		3.6 ± 2.2 ^3^		9.9 ± 4.8 ^3^	
4–5-year olds	59.6 ± 11.6 ^2^		28.6 ± 8.2		7.3 ± 2.8 ^1,2^		4.4 ± 2.3 ^1,2^		11.7 ± 4.8 ^1,2^	
*Sex*		0.374		0.613		**0.013**		**0.003**		**0.003**
Female	61.9 ± 11.6		28.6 ± 8.1		6.1 ± 2.7		3.3 ± 2.3		9.4 ± 4.8	
Male	60.9 ± 11.6		28.2 ± 8.3		6.8 ± 2.9		4.0 ± 2.7		10.9 ± 4.9	

Note. Data expressed as M ± SD. Boldface indicates statistical significance. Post hoc analysis (Tukey LSD): ^1^ Different from group 1. ^2^ Different from group 2. ^3^ Different from group 3.

**Table 3:** Upper-half of Median (Wear-time) Frequencies of Physical Activity and the Association with Age and Sex (*n* = 176).

	%Sedentary	*p*	%Light	*p*	%Moderate	*p*	%Vigorous	*p*	%MVPA	*p*
Overall	64.6 ± 7.9	NA	26.5 ± 5.8	NA	5.9 ± 2.1	NA	3.0 ± 1.5	NA	8.9 ± 3.4	NA
*Age (Years)*		**0.033**		0.889		**<0.001**		**<0.001**		**<0.001**
2-year olds	66.3 ± 7.1 ^3^		26.3 ± 5.7		5.1 ± 1.8 ^2,3^		2.3 ± 1.1 ^2,3^		7.4 ± 2.7 ^2,3^	
3-year olds	64.5 ± 7.2		26.4 ± 5.6		6.0 ± 1.9 ^1^		3.1 ± 1.1 ^1,3^		9.1 ± 2.8 ^1,3^	
4–5-year olds	62.6 ± 8.9 ^1^		26.8 ± 6.1		6.7 ± 2.3 ^1^		3.9 ± 1.8 ^1,2^		10.6 ± 3.9 ^1,2^	
*Sex*		0.123		0.780		**0.007**		**0.001**		**0.002**
Female	65.4 ± 8.5		26.4 ± 6.4		5.5 ± 2.0		2.7 ± 1.4		8.1 ± 3.3	
Male	63.6 ± 7.0		26.6 ± 5.0		6.3 ± 2.1		3.4 ± 1.5		9.7 ± 3.4	

Note. Data expressed as M ± SD. Boldface indicates statistical significance. Post hoc analysis (Tukey LSD): ^1^ Different from group 1. ^2^ Different from group 2. ^3^ Different from group 3.

**Table 4:** Comparison of Measures by Children’s Ethnicity and Weight Status.

	All	Hispanic	Non-Hispanic	*p*	Upper Median	Hispanic	Non-Hispanic	*p*
	(*n* = 342)	(*n* = 194)	(*n* = 140)		(*n* = 176)	(*n* = 91)	(*n* = 79)	
Demographics/Anthropometrics								
*Age (months)*	41.5 ± 11.8	41.3 ± 11.5	41.5 ± 12.0	0.918	40.8 ± 11.3	39.7 ± 10.5	42.5 ± 12.1	0.111
*Age (Years)*				0.614				0.191
2	122 (36)	69 (35)	56 (39)		67 (38)	37 (40)	28 (35)	
3	105 (31)	62 (32)	39 (27)		53 (30)	30 (34)	20 (25)	
4–5	115 (33)	64 (33)	50 (35)		56 (32)	24 (26)	32 (40)	
*Sex*				0.559				0.912
Female	179 (52)	100 (51)	79 (55)		92 (52)	44 (47)	38 (48)	
Male	163 (48)	95 (49)	66 (45)		84 (48)	48 (53)	41 (52)	
*Weight Status*				0.747				0.691
Non-Overweight	214 (65)	118 (65)	94 (66)		121 (68)	60 (69)	56 (72)	
Overweight	113 (35)	65 (35)	48 (34)		50 (32)	27 (31)	22 (28)	
*BMI* (kg/m^2^)	17.0 ± 2.1	16.9 ± 2.1	17.0 ± 2.1	0.802	16.8 ± 2.0	16.7 ± 1.7	16.8 ± 2.2	0.797
*BMI z-score* ^1^	0.7 ± 1.2	0.7 ± 1.2	0.7 ± 1.1	0.931	0.6 ± 1.1	0.6 ± 1.1	0.6 ± 1.1	0.872
*BMI Percentile*	66.5 ± 28.0	66.1 ± 28.6	66.9 ± 27.3	0.791	64.2 ± 27.4	64.7 ± 27.6	64.9 ± 26.5	0.958
*Waist Circumference*	53.1 ± 5.5	53.6 ± 5.7	52.2 ± 5.2	**0.035**	52.7 ± 5.0	53.1 ± 4.9	52.2 ± 4.9	0.221
Physical Activity								
*% Sedentary*	61.5 ± 11.1	62.5 ± 11.1	60.6 ± 11.9	0.317	64.6 ± 7.9	66.2 ± 8.3	62.6 ± 6.9	**0.007**
*% Light*	26.5 ± 5.8	27.6 ± 7.8	29.3 ± 8.7	0.074 ^†^	26.5 ± 5.8	25.4 ± 6.3	27.7 ± 4.9	**0.008**
*% Moderate*	6.4 ± 2.7	6.3 ± 2.8	6.6 ± 2.7	0.239	5.9 ± 2.1	5.5 ± 2.0	6.4 ± 2.2	**0.018**
*% Vigorous*	3.5 ± 2.4	3.6 ± 2.2	3.6 ± 2.8	0.797	3.0 ± 1.5	2.9 ± 1.4	3.2 ± 1.6	0.655
*% MVPA*	9.9 ± 4.7	9.9 ± 4.7	10.1 ± 4.9	0.642	8.9 ± 3.4	8.4 ± 3.2	9.6 ± 3.7	0.085 ^†^

Note. Data expressed as M ± SD or *n* (%). Boldface indicates statistical significance. ^†^ Approaching Significance. ^1^ Sex- and age-adjusted BMI z-score (<1 standard deviation = Non-overweight; ≥1 standard deviation = overweight). Analysis with all PA variables with full dataset adjusted for waist circumference. In addition, all analysis with %MPA, %VPA, and %MVPA adjusted for age and sex. Analysis with % Sedentary (upper median half) adjusted for age.

## Data Availability

The data presented in this study are available on request from the corresponding author. The data are not publicly available due to privacy and ethical considerations.
